# Climate Change Policies and Social Inequalities in the Transport, Infrastructure and Health Sectors: A Scoping Review Protocol

**DOI:** 10.3390/ijerph23010065

**Published:** 2025-12-31

**Authors:** Estefania Martinez Esguerra, Marie-Claude Laferrière, Anouk Bérubé, Pierre Paul Audate, Thierno Diallo

**Affiliations:** 1 Department of Geography, Université de Montréal, Montréal, QC H3C 3J7, Canada; estefania.martinez.esguerra@umontreal.ca (E.M.E.); pierre.paul.audate@umontreal.ca (P.P.A.); 2 Faculty of Nursing Sciences, Université Laval, Québec, QC G1V 0A6, Canada; marie-claude.laferriere@bibl.ulaval.ca (M.-C.L.); anouk.berube.1@ulaval.ca (A.B.); 3 VITAM—Centre de Recherche en Santé Durable, Québec, QC G1J 2G1, Canada

**Keywords:** climate change, public health, transport, infrastructure, social inequalities, OECD countries

## Abstract

**Highlights:**

**Public health relevance—How does this work relate to a public health issue?**

- This scoping review proposes a framework for assessing climate change adaptation and mitigation policies and their differentiated impacts on the public health and well-being of diverse population groups.

**Public health significance—Why is this work of significance to public health?**

- It aims to provide policymakers with evidence-based examples of promising initiatives that ensure vulnerable groups are considered in the design of climate policies, with the goal of informing the development of more just public health policies in the National Capital Region of Québec, Canada.

**Public health implications—What are the key implications or messages for practitioners, policy makers and/or researchers in public health?**

- It calls for integrating structural determinants of health inequalities into climate change policy design and implementation.

**Abstract:**

Climate action has been deemed as fundamental to counteract the impacts of rising global temperatures on health which will disproportionately affect low-income populations, racial and ethnic minorities, women, and other historically marginalized groups. Along with poverty reduction, inequality mitigation, gender equality promotion, and public health protection, climate action has been recognized as a fundamental goal for achieving Sustainable Development Goals (SDGs). However, despite growing recognition of the need to align climate action with development goals, there is a knowledge gap regarding how the implementation of climate change mitigation and adaptation policies impacts social inequalities. To address this knowledge gap, this document proposes a scoping review protocol aimed at identifying and synthesizing research that examines the impacts of climate policies on inequalities at the subnational scales, within the transport, infrastructure and health. The objective of this review is to map existing evidence, identify conceptual and empirical gaps and inform policy strategies that promote climate action in line with values of social justice and equality.

## 1. Introduction

Climate change represents not only a potential threat to the planet and its ecosystems, but also a significant challenge to efforts aimed at reducing social inequalities that affect global health. A global temperature rise exceeding 1.5 °C is expected to accelerate biodiversity loss and increase human disease and morbidity associated with exposure to extreme heat [1,2], as well as exacerbate social inequalities that further affect health outcomes. The World Bank [3] anticipates that the poorest and most vulnerable populations will be disproportionately affected by the impacts of climate change due to their location in geographic areas more exposed to extreme weather events, their financial, socioeconomic and socio-cultural conditions, and their limited access to resources, services and decision-making power. Groups at highest risk of suffering the environmental and social consequences of global temperature rise include low-income populations, ethnic and racial minorities, women, children, older adults, individuals with chronic diseases or disabilities, those living in areas prone to climate-sensitive diseases, as well as workers exposed to extreme heat [4].

The United Nations 2030 Agenda for Sustainable Development (SDGs) [5] includes climate action (SDG 13) alongside goals such as poverty alleviation (SDG 1), reducing inequality (SDG 10), promoting gender equality (SDG 5), and ensuring health and well-being for all (SDG 3) [5,6]. However, despite recognition of the need to align climate action with the achievement of specific goals such as reducing social inequalities, the interconnections between these objectives remain insufficiently interrogated [7]. For example, little attention has been paid to how the reduction in the carbon footprint relates to improving the living conditions of the most vulnerable groups. This gap is critical, as emerging evidence shows that while there are potential synergies between mitigation actions and the achievement of the SDGs, there are also significant trade-offs that can deepen social inequalities, heighten existing vulnerabilities, and widen disparities [8,9,10].

Recent studies have sought to address this gap by conceptualizing the distributional effects and social co-impacts of mitigation strategies. For example, Markkanen and Anger-Kraavi [10] show that the effects of climate policies on inequality depend heavily on context, policy design, and implementation. Similarly, Zahnow et al. [8] highlight how such policies can generate unequal outcomes across geographical and socioeconomic scales, underscoring significant cross-country differences. However, while these analyses focus primarily on policy implementation at the global and national levels, they often overlook the social dynamics within subnational scales as well as structural factors—such as socioeconomic status, gender, or ethnicity—that shape social inequalities in climate change adaptation and mitigation [11]. Although a growing body of literature recognizes the importance of integrating these geographical and social scales within a broader understanding of health inequalities [12], exhaustive analyses of subnational climate change policy implementation and their differential effects on the configuration of social inequalities are still limited.

This scoping review aims to provide a systematic mapping of subnational climate policy and their differentiated impact on inequalities related to gender, ethnicity, and income. Subnational mitigation and adaptation policies refer to the plans, strategies and programs implemented by regional and local governments, including cities and municipalities—with the aim of reducing or preventing greenhouse gas (GHG) emissions and decreasing vulnerability to the current or projected impacts of climate change in different sectors like energy, infrastructure, and health [13,14]. Subnational policies may include land-use planning tools (such as zoning and urban densification plans); strategies and programs designed to reduce GHG emissions in urban transport (e.g., carbon taxes); the decarbonization of residential and commercial buildings; heat-risk adaptation plans for hospitals and buildings; and response plans for extreme climate events such as heatwaves and floods [15,16].

This analysis focuses on transport, infrastructure (buildings), and health, identified by the Intergovernmental Panel on Climate Change (IPCC) Working Group II as key areas for climate change mitigation and adaptation [17,18]. Within the transport sector, the analysis concentrates primarily on collective transport, with particular attention to urban mobility. We focused on urban transport because more than half the world’s population (over 4 billion people around the world) live in cities (World Bank 2025) [19]. In the infrastructure and health sectors, the review concentrates on residential buildings and hospitals, respectively. These sectors hold dual importance: not only are they vital for limiting greenhouse gas (GHG) emissions, but they also play a key role in mitigating the population’s vulnerability to climate change effects. Transport and buildings together account for approximately 50% of global energy demand and nearly 70% of global GHG emissions [20,21]. According to the United Nations Environment Programme (2025) [20], in 2023, the building and construction sector accounted for 32 percent of global energy consumption. Buildings alone are responsible for about 31% of global carbon dioxide emissions, with roughly half attributable to energy use in residential buildings [22], while the health sector accounts for approximately 5% of global emissions [23].

Interventions in these sectors offer significant opportunities to reduce emissions while also addressing structural social inequalities related to housing conditions, access to urban transport, and exposure to environmental risks—factors that disproportionately affect populations according to socioeconomic status, ethnicity, and gender. Housing is central to addressing the health impacts of climate change and to addressing structural conditions of inequality [22]. Policies aimed at reducing emissions from residential heating and cooling, as well as energy-efficiency retrofits in housing, can help mitigate long-standing inequalities linked to spatial segregation and chronic underinvestment in infrastructure, while also reducing household energy costs—a burden that disproportionately affects low-income households. Similarly, improvements in collective transport systems can reduce emissions from private vehicle use while enhancing mobility for low-income households in underserved neighborhoods, which typically have lower car ownership yet experience higher exposure to traffic-related pollution and environmental harm due to urban sprawl and socio-spatial polarization [24].

This research protocol will examine the extent to which climate actions implemented within these sectors recognize and address social inequalities related to socioeconomic status, ethnicity, and gender. In doing so, the review seeks not only to advance a critical understanding of the social dimensions of climate change mitigation and adaptation policies, but also to inform the development of more just public health policies in the National Capital Region of Québec, Canada. The overarching objective is to provide policymakers with evidence-based examples of promising initiatives capable of ensuring that vulnerable groups are adequately considered in the design of climate policies. To ensure the relevance and comparability of the policies reviewed to the Québec–Canada context, the scope of this protocol will be limited to high-income OECD countries—such as Denmark, Finland, France, Iceland, Japan, Korea, New Zealand and the United Kingdom. These countries share similar governance structures, institutional capacities, and socioeconomic conditions. The selected policies will be systematically classified according to their level or scale of implementation (provincial/state, municipal, sectoral/neighborhood), the sector targeted (infrastructure, transport, health), the type of mitigation or adaptation measure employed, and the dimension of inequality addressed (race/ethnicity, income, gender). This structured analytical framework will facilitate the identification of patterns, divergences, and best practices across contexts, while ensuring comparability across countries and policy domains.

## 2. Materials and Methods

### 2.1. Study Design

Given the broad scope of the topic and the dispersed nature of the existing literature across multiple disciplines—including public health, climatology, geography, urban planning, energy studies, and transportation—this study will examine the intersection of climate change policies and social inequalities through a scoping review of the literature. Unlike the systematic review, which focuses on assessing the effectiveness of interventions and the strength of evidence, the scoping review is well suited to mapping the breadth of available evidence and identifying gaps in knowledge [25]. Carrying out a scoping review within this research will be useful to identify and describe the type of evidence that addresses the relationship between climate change policies and social inequalities and the way the research is conducted.

Methodologically, this scoping review will follow the framework developed by Arksey and O’Malley [26] and later refined by Levac et al. [27], which outlines six essential steps for conducting scoping reviews: (1) Identifying the research question (2) Searching for relevant studies (3) Selecting studies (4) Charting the data (5) Collating, summarizing, and reporting the results, and (6) Consulting with stakeholders to inform or validate the study’s findings. In addition, the review will adhere to the PRISMA-ScR (Preferred Reporting Items for Systematic Reviews and Meta-Analyses extension for Scoping Reviews) checklist as recommended by Tricco et al. [28] and it will be published rather than registered.

### 2.2. Identifying the Research Question

A central question in this scoping review protocol is: To what extent do climate change adaptation and mitigation policies in the urban transport, infrastructure (buildings), and health sectors contribute to reducing or exacerbating social inequalities related to income, gender, and ethnicity?

The research aims to analyze the extent to which the implementation of sectoral climate change mitigation and adaptation policies has an impact on social inequalities. Inequalities are conceptualized as systemic injustices that prevent or limit certain individuals or groups from accessing public goods, resources, and opportunities on equal terms [29]. These inequalities may be associated with structural factors such as income, gender, race, and ethnicity. They are exacerbated by social conditions such as inadequate housing, energy poverty, or limited access to essential services. The study examines how these inequalities are assessed in existing research and policy documents on climate change, particularly regarding their positive or negative impacts on low-income populations, women, ethnic minorities, and racialized groups. Guided by the Population, Concept, and Context (PCC) framework recommended by the Joanna Briggs Institute (JBI), this study will identify racial minorities and low-income groups as key target populations of climate change adaptation and mitigation policies. The analysis will also be limited to the context of high-income OECD countries (see Table 1).

### 2.3. Search Strategy

The search strategy will focus on bibliographic information and abstracts in the fields of biomedicine and pharmacology; life sciences; social sciences, education, and psychology; geographical, earth, and ecological sciences; and other multidisciplinary areas, as well as on policy and gray literature. These disciplinary areas are not weighted equally; rather, the aim is to capture literature from all relevant fields—public health, climatology, geography, urban planning, energy, and transportation—only insofar as it analyses climate policies and their impacts on social inequalities. The search will be conducted in five bibliographic databases: (1) Medline (OvidSP), (2) Embase.com, (3) Academic Search Premier (EBSCOHost), (4) Geobase (Engineering Village), and (5) Web of Science (SCI-EXPANDED, SSCI, AHCI, ESCI, CPCI-S, CPCI-SSH)—as well as two policy and gray literature databases: (1) Policy Citation Index (Clarivate) and (2) Overton.io.

The strategy will combine free-text terms and controlled vocabulary related to “climate change”, “adaptation and mitigation policies” and terms related to “social inequity”—such as “economically disadvantaged populations” and “ethnic minorities” (See Table 2). It should be noted that the search strategy may not identify studies assessing the effectiveness of specific measures to reduce CO_2_ emissions, GHG emissions, F-gas emissions, or similar outcomes unless they explicitly reference climate change or social inequalities. A modified version of the OECD country geographic search filters from the NICE program will be applied [30]. The results of this scoping review will be compiled and imported into the Covidence software. Duplicates will be removed.

### 2.4. Eligibility Criteria

The selection of studies will be guided by the inclusion and exclusion criteria established by the research team, in alignment with the search strategy. The aim of the strategy is to identify studies and policies on climate change adaptation and mitigation in key sectors—transport, infrastructure (buildings), and health—within OECD countries, with a focus on their impacts on social inequalities. Studies outside these sectors, or those that do not explicitly link climate action to social inequality will be excluded. This approach ensures that the review remains focused on policies and interventions relevant to our research objectives while maintaining clear boundaries around the included evidence.

In the transport sector, climate change mitigation and adaptation actions may include:

- Policies aimed at reducing GHG emissions from public transportation.
- Managing transport demand through land-use planning and policy instruments.
- Programs promoting low-emission modes of transport.
- Policies encouraging the adoption of energy-efficient vehicle technologies.
- Land-use planning and density regulations.

In the building sector, particularly the residential sub-sector, mitigation and adaptation policies may include:

- Energy-efficiency retrofits in homes (e.g., reducing energy consumption for heating and cooling).
- Legislation promoting or mandating the use of low-carbon construction materials.
- Municipal programs facilitating the transition from fossil fuel-based heating and cooling systems to electric alternatives.
- Financial incentives for the adoption of solar photovoltaic (PV) systems in buildings and homes.
- Heat- and extreme weather-adaptation plans.

In the health sector, actions may include:

- Public policies promoting energy conservation in healthcare facilities.
- Implementation of heat-risk adaptation measures in hospitals.
- Protection of high-risk and vulnerable populations.
- Preparedness for climate-related risks.
- Policies enhancing local capacities to respond to extreme weather events.

Studies focused on the agricultural, industrial, or extractive sectors will be excluded. Although activities related to food production, energy production, and natural resource extraction significantly shape social inequalities, research on adaptation and mitigation policies in these sectors generally focuses on regions of the Global South and falls outside the scope of this review. Similarly, studies examining adaptation or mitigation policies in the downstream energy sector (i.e., production) will be excluded, whereas studies on climate policies in the upstream energy sector—particularly those related to distribution and consumption that directly intersect with infrastructure (e.g., buildings) and transport systems—will be included.

The selection of studies will focus on original studies based on qualitative or quantitative research, modeling studies or literature review. Theoretical and conceptual debates will be included only if they offer a substantive analysis of climate policies (e.g., laws, policy frameworks) and their implications for social inequalities; philosophical or normative debates and commentaries will be excluded. Although these discussions are important, they fall outside the specific scope and objectives of this review. Finally, to ensure comparability between climate change policies and the research results, studies focusing on low-middle-income and non-OECD countries will be excluded (see Table 3).

#### Gray Literature

The primary selection criterion for gray literature documents is that they refer to climate policies or plans implemented by governments, institutions, and organizations at the local, national, and international levels (see Table 4). This includes reports and documents produced by international organizations such as the Intergovernmental Panel on Climate Change (IPCC), the United Nations Development Programme (UNDP), the United Nations Environment Programme (UNEP), the World Bank, the Organisation for Economic Co-operation and Development (OECD), among others. These documents typically explore the relationship between climate policies and social inequalities, as well as their implications for equity and climate justice. Documents published by for-profit entities (e.g., corporate mitigation plans), documents published in blogs, or press releases by non-governmental organizations, will be excluded. Likewise, documents that deal with climate change but do not address social inequalities will be excluded.

### 2.5. Selection Process

Four members of the research team (E.M.E., A.B., P.P.A, and T.D), in collaboration with a librarian (M.-C.L.), will carry out the scoping literature search. The team will meet to conclude and thoroughly discuss the inclusion and exclusion criteria that will guide the selection of studies aligned with the research question and objectives. Subsequently, each reviewer will screen the citation and abstract fields of bibliographic databases using Covidence software. Studies meeting these criteria will proceed to the full-text review stage. At this stage, two independent reviewers will assess the full texts of potentially relevant studies. In cases of disagreement between reviewers, a third team member will be consulted to make the final inclusion or exclusion decision.

Simultaneously, the citation and abstract fields of gray literature from the combined Policy Citation Index and Overton.io databases will be exported in Excel format. Two reviewers will independently screen these citations using predefined inclusion and exclusion criteria. Duplicates will be identified manually and highlighted, and a column adjacent to each reference will indicate whether the document is considered relevant. Any disagreements will be resolved by a third reviewer.

Finally, the selected citations and abstracts from both bibliographic and gray literature sources will be compiled into a single Excel sheet.

### 2.6. Data Charting Process

Data will be extracted using Covidence. In addition to basic study information—such as author(s), publication date, study type, geographic scale (national, regional, local/city/, neighborhood, household), and country or region of implementation—data will also be collected on the type of mitigation or adaptation policy, the sector of implementation, the identification of population groups by type of inequality, and the observed impacts on these inequalities. Observed impacts will be classified as positive when policies result in a reduction in vulnerabilities related to socioeconomic, gender, or racial status. For example, when they lead to:

- Greater inclusion and empowerment of marginalized groups;
- Enhanced participation in decision-making processes;
- Improved quality of life and well-being;
- Increased access to services (e.g., transport, energy, healthcare, housing);
- Job creation and local economic opportunities;
- Improved health outcomes and reduced exposure to environmental risks;
- Reduced energy costs or improved energy efficiency for low-income households;
- Strengthened social cohesion;
- Greater gender equity and representation of women in climate governance;
- Recognition of Indigenous rights and knowledge;
- Co-benefits for health and the environment (e.g., cleaner air, creation of green spaces).

Conversely, impacts will be classified as negative when policies exacerbate socioeconomic, gender-based, or racial inequalities and vulnerabilities. For example, when they cause:

- Limit access or restrict benefits for certain groups;
- Displacement or gentrification;
- Job losses in specific sectors;
- A higher cost of living (e.g., increase the cost of energy, transportation, and housing);
- Unequal participation in decision-making or gender bias;
- Exclusion or marginalization of vulnerable, Indigenous, or racialized populations;
- Exposure to new or aggravated risks due to poorly designed measures (e.g., shifting policy burdens to consumers rather than major emitters).

Finally, data will also be extracted on the recommendations proposed by studies or policies to reduce social inequalities associated with the implementation of climate change mitigation and adaptation measures. Extracted data will be compiled and organized in an Excel spreadsheet to facilitate data synthesis and management (see Table 5).

### 2.7. Collating, Summarizing and Reporting the Results

Following the data extraction phase, two reviewers will independently review, interpret, and code the relevant data using the qualitative analysis software NVivo 15. A coding framework will be developed both a priori and iteratively as the inductive analysis progress. Any discrepancies between reviewers will be resolved through discussion, with a third reviewer consulted if necessary. Descriptive numerical summaries (e.g., number of studies by year, country, and policy type) will be presented using tables and figures to provide a clear overview of the characteristics of the included studies. In addition, an inductive analysis will be conducted to identify key patterns, emerging themes, and knowledge gaps related to the impacts of climate change mitigation and adaptation policies on social inequalities.

Given the exploratory nature of the study, we expect the results to be heterogeneous and wide-ranging. As this is a scoping review—and not a systematic review requiring a formal risk of bias assessment—no quality appraisal will be conducted. Scoping reviews aim to map the extent, range and nature of existing research on a broad topic. This approach aligns with our research objective, which is to map the breadth and nature of existing subnational climate policies that address social inequalities, rather than to evaluate the effectiveness or methodological rigor of individual studies. By focusing on identifying, categorizing, and synthesizing the range of policy actions reported in the literature, the scoping review methodology will enable us to capture the full extent of the evidence base, including emerging, heterogeneous, or conceptually oriented studies that might not meet the criteria for a systematic quality assessment. The final synthesis will highlight areas for future research and provide policy-relevant insights aimed at reducing social inequalities through climate action. The final synthesis will highlight areas for future research and provide policy recommendations aimed at reducing social inequalities through climate policy actions.

### 2.8. Stakeholder Consultation to Support or Validate Findings

The findings of the scoping review will be examined and discussed in collaboration with a group of academic and policy experts, including representatives from the Quebec Public Health Department (DSPu-CN), the Institut national de santé publique du Québec (INSPQ), the National Collaborating Centre for Healthy Public Policy (NCCHPP) and four university-affiliated researchers. The INSPQ is a governmental institution that works closely with Québec’s health and social services network, while the NCCHPP is a publicly funded center dedicated to strengthening Canada’s public health infrastructure. These institutions were selected as primary stakeholders due to their recognized expertise and their involvement in areas of public health policy that intersect directly with the thematic scope of this research. Stakeholders will contribute not only to validating the findings but also to interpreting and prioritizing emerging themes and policy implications. This collaborative process aims to support the co-creation of a typology of co-benefits associated with climate change adaptation and mitigation actions, policies, and strategies across the targeted sectors, and to assess their relevance and potential impact within both the regional (Québec) and national (Canadian) contexts. Overall, this consultation phase will enhance the methodological transparency and rigor of the review. Since these public institutions are not involved in the funding of the project and their role is limited to external consultation and validation, no conflicts of interest are anticipated.

The insights obtained from stakeholder consultations will be synthesized and incorporated into a dedicated recommendations section of the scoping review. In addition to the variables analyzed in the scoping review, this section will integrate stakeholders’ assessments of which climate policies and interventions appear most applicable and transferable to the Québec–Canada context. Stakeholders’ perspectives will be used to contextualize the findings, identify context-specific considerations—including feasibility, equity impacts, and implementation challenges—and refine the relevance of the policy examples for regional and national decision-making.

## 3. Conclusions

This scoping review protocol proposes a systematic approach to mapping existing research on the relationship between climate change mitigation and adaptation policies and social inequalities in OECD countries. While a substantial body of literature examines the impacts of climate change on social inequalities, there is limited evidence on the effects of subnational adaptation and mitigation policies on social inequalities.

One of the underlying hypotheses of this research is that a gap exists between studies and policies focused on climate change mitigation and adaptation and those explicitly aimed at reducing social inequalities. This gap may stem from the fact that much of the literature concentrates on the global or national scale, while the effects of these policies at subnational and local levels remain underexplored [8]. Moreover, the effectiveness of climate policies is often evaluated primarily in terms of environmental outcomes—such as emissions reductions—while their social equity implications are overlooked.

Potential findings may reveal both synergies and trade-offs between climate policies and the reduction in social inequalities, depending on the scale and design of implementation. Policies that fail to adequately reach low-income or marginalized communities, or that prioritize aggregate national outcomes over local realities, may inadvertently exacerbate social inequities. Likewise, measures such as carbon taxes or urban and land-use planning regulations could unintentionally disadvantage low-income groups if accessibility and affordability are not considered. Conversely, policies that explicitly target vulnerable groups—such as women, racialized populations, Indigenous peoples, and economically disadvantaged communities—have the potential to reduce inequality gaps while simultaneously advancing climate objectives. Therefore, the results of this review could help identify promising practices to limit the negative impacts of policies on the deepening of social inequalities.

## Figures and Tables

**Table 1:** Inclusion criteria based on the PCC framework (population, concept, and context).

PCC Element	Example
Population	Low-income groups, racialized groups/ethnic minorities
Concept	Implementation of climate change mitigation and adaptation policies and the reduction or exacerbation of social inequalities
Context	High-income OECD countries

**Table 2:** Example of search strategy.

#	Query
1	climate change mitigation’/de OR ‘flood mitigation’/de
2	climate change*’:ti,ab,kw OR ‘global warming’:ti,ab,kw OR ‘climate crisis’:ti,ab,kw OR ‘climate warming’:ti,ab,kw OR ‘climate issue*’ OR ‘climate vulnerabilit*’:ti,ab,kw OR ‘climate emergenc*’:ti,ab,kw OR ‘heat-risk’:ti,ab,kw OR ‘extreme heat’:ti,ab,kw OR ‘heat wave*’:ti,ab,kw OR heatwave*:ti,ab,kw OR ‘extreme weather’:ti,ab,kw OR ‘extreme hot weather’:ti,ab,kw
3	climate change’/exp OR ‘extreme weather’/exp OR ‘heat wave’/exp OR flood:ti,ab,kw OR flooding:ti,ab,kw OR ‘flooding’/de
4	#2 OR #3
5	adaptat*:ti,ab,kw OR mitigat*:ti,ab,kw
6	adaptation’/de OR ‘mitigation’/de
7	#5 OR #6
8	action*:ti,ab,kw OR initiative*:ti,ab,kw OR plan OR plans OR planning OR policies OR policy OR practice*:ti,ab,kw OR program*:ti,ab,kw OR strateg*:ti,ab,kw
9	‘policy’/exp
10	#8 OR #9
11	#4 AND #7 AND #10
12	(climate NEAR/7 (action* OR initiative* OR measures OR measure OR plan OR plans OR planning OR policies OR policy OR program* OR projet OR projets OR transition OR transformation OR strateg*)):ti,ab,kw
13	((‘low carbon’ OR decarbonization OR decarbonisation) NEAR/7 transition*):ti,ab,kw
14	#1 OR #11 OR #12 OR #13
15	inequalit*:ti,ab,kw OR inequit*:ti,ab,kw OR disparit*:ti,ab,kw OR injustice:ti,ab,kw OR unfairness:ti,ab,kw OR equit*:ti,ab,kw OR equalit*:ti,ab,kw OR justice:ti,ab,kw OR fairness:ti,ab,kw OR ‘just transition’:ti,ab,kw
16	‘social inequality’/exp OR ‘social justice’/exp OR ‘health equity’/de
17	low income’:ti,ab,kw OR ‘lower income’:ti,ab,kw OR poverty:ti,ab,kw OR ‘economically disadvantage*’:ti,ab,kw OR ((poor NEAR/3 (working OR people OR man OR men OR women OR woman OR population* OR habitant* OR neighborhood*)):ti,ab,kw)
18	lowest income group’/exp OR ‘poverty’/exp
19	gender:ti,ab,kw OR genders:ti,ab,kw
20	‘gender’/de
21	ethnicit*:ti,ab,kw OR race OR racial:ti,ab,kw OR ‘ethnic minorit*’:ti,ab,kw OR ‘ethnic group*’:ti,ab,kw
22	ethnic or racial aspects’/exp OR ‘minority group’/de OR ‘ethnic group’/de
23	‘first nation’:ti,ab,kw OR ‘first nations’:ti,ab,kw OR ‘first people*’:ti,ab,kw OR amerindian*:ti,ab,kw OR autochtone*:ti,ab,kw OR aborig*:ti,ab,kw OR (((native* OR indigenous) NEAR/1 (american OR man OR men OR women OR woman OR person* OR adult OR people* OR indian* OR nation OR tribe* OR tribal OR band OR bands OR communit*)):ti,ab,kw)
24	‘indigenous people’/exp OR ‘metis’/de OR ‘eskimo’/de OR ‘inuit’/de
25	#15 OR #16 OR #17 OR #18 OR #19 OR #20 OR #21 OR #22 OR #23 OR #24
26	#4 AND #10 AND #25
27	‘arctic and antarctic’/exp OR ‘central asia’/exp OR ‘china’/exp OR ‘north korea’/de OR ‘mongolia’/de OR ‘philippines’/de OR ‘southeast asia’/exp OR ‘taiwan’/de OR ‘bahrain’/de OR ‘cyprus’/de OR ‘iran’/de OR ‘iraq’/exp OR ‘jordan’/de OR ‘kuwait’/de OR ‘lebanon’/de OR ‘united arab emirates’/exp OR ‘oman’/de OR ‘palestine’/de OR ‘qatar’/de OR ‘saudi arabia’/de OR ‘syrian arab republic’/de OR ‘yemen’/de OR ‘northern asia’/de OR ‘south asia’/exp OR ‘egypt’/de OR ‘georgia (republic)’/de OR ‘africa’/exp OR ‘albania’/de OR ‘armenia’/de OR ‘azerbaijan’/exp OR ‘balkan peninsula’/de OR ‘belarus’/de OR ‘bosnia and herzegovina’/exp OR ‘bulgaria’/de OR ‘croatia’/de OR ‘georgia (republic)’/exp OR ‘kosovo’/de OR ‘moldova’/de OR ‘montenegro (republic)’/de OR ‘republic of north macedonia’/de OR ‘romania’/de OR ‘russian federation’/exp OR ‘serbia’/exp OR ‘ukraine’/exp OR ‘andorra’/de OR ‘gibraltar’/de OR ‘malta’/de OR ‘san marino’/de OR ‘vatican city state’/de OR ‘liechtenstein’/de OR ‘monaco’/de OR ‘azores’/de OR ‘bermuda’/de OR ‘bouvet island’/de OR ‘canary islands’/de OR ‘caribbean islands’/exp OR ‘falkland islands (malvinas)’/de OR ‘greenland’/de OR ‘madeira’/de OR ‘saint helena’/de OR ‘saint pierre and miquelon’/de OR ‘sao tome and principe’/de OR ‘south georgia and the south sandwich islands’/de OR ‘svalbard and jan mayen’/de OR ‘belize’/de OR ‘caribbean’/exp OR ‘el salvador’/de OR ‘guatemala’/de OR ‘honduras’/de OR ‘nicaragua’/de OR ‘panama’/de OR ‘argentina’/de OR ‘aruba’/de OR ‘bolivia’/de OR ‘brazil’/exp OR ‘caribbean netherlands’/exp OR ‘ecuador’/de OR ‘french guiana’/exp OR ‘guyana’/de OR ‘netherlands antilles’/de OR ‘paraguay’/de OR ‘peru’/de OR ‘suriname’/de OR ‘uruguay’/exp OR ‘venezuela’/exp OR ‘indian ocean’/exp OR ‘pacific islands’/exp
28	‘organisation for economic co-operation and development’/de OR ‘european union’/de OR ‘developed country’/de
29	‘australia and new zealand’/exp OR ‘austria’/de OR ‘baltic states’/exp OR ‘belgium’/exp OR ‘chile’/de OR ‘colombia’/de OR ‘costa rica’/de OR ‘czech republic’/de OR ‘denmark’/de OR ‘europe’/de OR ‘finland’/exp OR ‘france’/exp OR ‘germany’/exp OR ‘greece’/de OR ‘hungary’/de OR ‘iceland’/de OR ‘ireland’/de OR ‘israel’/de OR ‘italy’/exp OR ‘japan’/de OR ‘korea’/de OR ‘luxembourg’/de OR ‘netherlands’/de OR ‘north america’/exp OR ‘norway’/exp OR ‘poland’/de OR ‘portugal’/exp OR ‘scandinavia’/de OR ‘slovakia’/de OR ‘slovenia’/de OR ‘south korea’/de OR ‘spain’/exp OR ‘sweden’/de OR ‘switzerland’/de OR ‘turkey (republic)’/exp OR ‘united kingdom’/exp OR ‘western europe’/de
30	#28 OR #29
31	#27 NOT #30
32	#26 NOT #31

**Note:** Rows #1–#4 capture climate change and related hazards using controlled vocabulary and free-text terms. Rows #5–#7 focus on adaptation and mitigation, while rows #8–#10 cover policy-related terms. Rows #11–#14 combine these groups using Boolean and proximity operators to identify studies on climate-related actions or interventions. Rows #15–#25 address social inequalities (e.g., income, gender, ethnicity, Indigenous peoples, poverty), and row #26 combines climate action/policy terms with social inequality terms. Rows #27–#31 apply geographic filters to include only high-income OECD countries and exclude low- and middle-income countries. Row #32 defines the final search, capturing studies on climate change, policy/actions, and social inequalities in relevant high-income contexts.

**Table 3:** Other inclusion and exclusion criteria.

Inclusion Criteria	Exclusion Criteria
Written in English or French	Not written in English or French
Empirical qualitative or quantitative study, modeling studies, literature reviews	Philosophical and normative debates
Theoretical and conceptual debates (e.g., laws, policy frameworks)	Commentary
Study focuses on the transport, infrastructure (buildings), health sectors	Study focuses on a sector other than transport, infrastructure (buildings) or health (e.g., the agriculture and land-use sector)
Study focuses on the energy sector (direct impact on the transport and infrastructure sector)	Reference area of the study is a low-middle-income and non-OECD member country
Reference area of the study is a high-income and OECD member country	

**Table 4:** Inclusion and exclusion criteria for gray literature.

Inclusion Criteria	Exclusion Criteria
Written in English or French	Not written in English or French
Public policy document (e.g., climate plans from local, national governments; reports from national and international organizations)	Mitigation plans of companies or for-profit organizations
Public policy analysis	Documents published in blogs of non-governmental organizations
Document focuses on climate change policies and their impacts on social inequalities	Press releases
Document focuses on transport, buildings, health or on a cross-cutting sector that impacts social inequalities in these sectors	Document focuses on climate change but does not address the issue of social inequalities
Reference area of the study is a high-income and OECD member country	Document focuses on a sector other than transport, buildings or health (e.g., the industry or the agricultural sector)
	Reference area of the study is a low-middle-income and non-OECD member country

**Table 5:** Data extraction table.

Category	Description		
Journal			
Publication Date			
Study type			
Geographic scale	National, regional	Local/city/, neighborhood	Household/individual
Country or region of implementation			
Type of Mitigation or Adaptation Policy			
Sector of Application	Infrastructure	Transport	Health
Population groups by type of inequality	Socioeconomic or income inequalities: Income: Populations affected by low Income, poverty, or economic disadvantage/ Communities experiencing financial constraints	Gender inequalities: Women, gender minority groups	Ethnicity and racial inequalities: Racialized groups/Ethnic Minority/Indigenous/ First Nations/marginalized ethnic groups
Observed Impacts on Social Inequalities	Inclusion and empowerment/Enhanced participation in decision-making processes/ Improved quality of life and well-being/ Increased access to services (transport, energy, healthcare, housing)/ Job creation/Local economic opportunities/ Improved health outcomes Reduced exposure to environmental risks/ Reduced energy costs or improved energy efficiency/ Social cohesion/Greater representation of women/ Recognition of Indigenous rights and knowledge/ Co-benefits for health and environment (e.g., cleaner air, green spaces)	Limited access to new infrastructures or technologies/Low affordability of adaptation or mitigation measures/Displacement or gentrification/ Job losses/ Increased costs of living (e.g., energy, transport, housing)/ Social exclusion of vulnerable or low-income groups/ Gender bias or unequal participation in decision-making/ Marginalization of Indigenous or racialized communities/ Health risks/Policy burden shifted to consumers rather than large emitters	
Policy or study recommendations to reduce social inequalities			

## Data Availability

The data that supports the findings of this study will be available on request from the corresponding author, T.D.

## Abbreviations

The following abbreviations are used in this manuscript:

IPCC	Intergovernmental Panel on Climate Change
GHG	Global Greenhouse Gas
SDGs	Sustainable Development Goals
OECD	Organisation for Economic Co-operation and Development
JBI	Joanna Briggs Institute
PRISMA-ScR	Preferred Reporting Items for Systematic Reviews and Meta-Analyses for Scoping Reviews
